# Xylo-oligosaccharides and virginiamycin differentially modulate gut microbial composition in chickens

**DOI:** 10.1186/s40168-015-0079-4

**Published:** 2015-04-10

**Authors:** Mohsen Pourabedin, Leluo Guan, Xin Zhao

**Affiliations:** Department of Animal Science, McGill University, 21111 Lakeshore Road, Ste-Anne-De-Bellevue, Quebec Canada; Department of Agricultural, Food and Nutritional Science, University of Alberta, 116 St. and 85 Ave., Edmonton, Alberta Canada

**Keywords:** Chicken, Gut microbiome, Xylo-oligosaccharides, Virginiamycin, Pyrosequencing

## Abstract

**Background:**

The emergence and spread of antibiotic resistance in pathogens have led to a restriction on the use of antibiotic growth promoters (AGPs) in animal feed in some countries. The potential negative after-effects of a ban on AGPs could be mitigated by improving animal intestinal health with prebiotic dietary fibers such as xylo-oligosaccharides (XOS). However, the mechanism(s) by which an antibiotic or prebiotic contributes to the health and growth of animals are not well understood. Here, we evaluated XOS and virginiamycin (VIRG)-mediated changes in gut microbiota of broiler chickens using pyrosequencing of the 16S rRNA gene.

**Results:**

There was a significant change in the relative abundance of certain bacteria, but the overall microbial diversity was not affected by treatment with either XOS or VIRG. Supplementation of HXOS (2 g XOS/kg diet) increased the proportion of *Lactobacillus* genus in the cecum, whereas *Propionibacterium* and *Corynebacterium* genera were enriched in the ileum of VIRG (16 mg/kg) treated birds. Furthermore, an increase in the cecal concentrations of acetate and propionate was observed in HXOS- and VIRG-fed chickens, respectively. These two groups of birds had better feed conversion efficiencies in comparison with the control group from day 7 to 21. In addition, temporal variations in the gut microbiota were evident in the chickens of different ages.

**Conclusions:**

Treatments with XOS or VIRG modified the relative abundance but not the presence or absence of specific microbial genus. The increase in both *Lactobacillus* spp. and acetate production in the cecum of HXOS-treated chickens may promote intestinal health.

**Electronic supplementary material:**

The online version of this article (doi:10.1186/s40168-015-0079-4) contains supplementary material, which is available to authorized users.

## Background

Antibiotic growth promoters (AGPs) have been widely used in poultry production to improve growth performance, feed efficiency, and overall health [1]. However, this practice has been discontinued in the European Union since 2006 due to increasing concern over spread of antibiotic-resistant genes to human and animal pathogens [2]. The withdrawal of AGP from poultry feed may increase bird disease rates, causing a rise in veterinary use of antibiotics for therapeutic purpose [3]. Therefore, there is the need to find effective alternatives to AGPs that improve chicken health and maintain efficiency of production and safety of poultry products.

Although it is still unclear how AGPs enhance animal performance, it is believed that they mainly act on gut microbiota [4]. The chicken gastrointestinal microbiota harbors dynamic and complex bacterial communities with an important role in metabolic activity and immune development [5]. In addition, some gut microbes produce a variety of enzymes that digest complex polysaccharides into short chain fatty acids (SCFA), mainly acetate, propionate, and butyrate. SCFA are major end products of bacterial fermentation of dietary fiber and provide several health benefits to the host including regulation of intestinal inflammation [6,7]. We have previously shown that supplementation of certain indigestible but fermentable dietary fibers, such as mannan oligosaccharides, promoted the growth of bacterial species with potential health benefits [8]. Accumulating evidence has suggested that xylo-oligosaccharides (XOS) are another promising prebiotic candidate [9,10]. While XOS are not degraded by recognized enteric pathogens such as *Staphylococcus aureus*, *Clostridium difficile*, *Salmonella enterica*, and *Campylobacter jejuni*, probiotic strains such as *Lactobacillus* spp. and *Bifidobacterium* spp. are able to utilize XOS [11,12]. However, the effect of XOS on the gut microbiota remains unclear as previous studies have often relied on *in vitro* observation [13-15] or microbial culture methods [16] that fail to provide accurate taxonomic composition and community structure.

Previous analysis of chicken intestinal microbiota using 16S rRNA clone library sequencing method has indicated that dietary supplementation with sub-therapeutic level of tylosin [17] or virginiamycin [18] influence the population of specific bacterial species in the small intestine. A pyrosequencing analysis of the 16S V3 region has also revealed a number of significant changes in the cecal microbiota of chickens treated with monensin in the presence or absence of tylosin or virginiamycin [19]. However, their study did not elucidate how virginiamycin alone could affect microbial communities in the cecum and other gastrointestinal tract locations. Far less is known about the prebiotic-mediated changes in the chicken microbiome.

We hypothesized that, in chicken, the mode of action of virginiamycin or XOS occurs through the gut microbiota. Therefore, it is necessary to better understand how gastrointestinal bacterial communities react to these feed additives. In this study, we used high-throughput sequencing of the V1-V3 region of 16S rRNA gene to assess the ileal and cecal microbiota in male broiler chickens fed either a commercial diet free of antibiotics and prebiotics (CTL), the same basal diet supplemented with a sub-therapeutic level (16.5 g/ton diet) of virginiamycin (VIRG), or the basal diet supplemented with 1 g/kg (LXOS) or 2 g/kg (HXOS) of XOS. Ileal and cecal concentrations of lactate and SCFA were also measured.

## Results

### Sequence analysis and quality filtering

A total of 2,063,514 pyrosequencing reads were obtained from 96 ileal and cecal samples. After removing 280,537 low-quality and chimeric sequences, the average number of reads generated per chicken was 17,570 (±8,459 STD) from ileal samples and 19,444 (±4,273 STD) from cecal samples, with the median read length of 402 (±93 STD) bases in all samples. In total, 6,544 distinct operational taxonomic units (OTUs) at the 97% identity level were obtained from all samples. After rare OTUs (<0.005% of total OTUs) were removed, a total of 3,248 OTUs remained for downstream analyses.

### Effects of dietary treatments on the ileal microbiota

**Figure 1:**
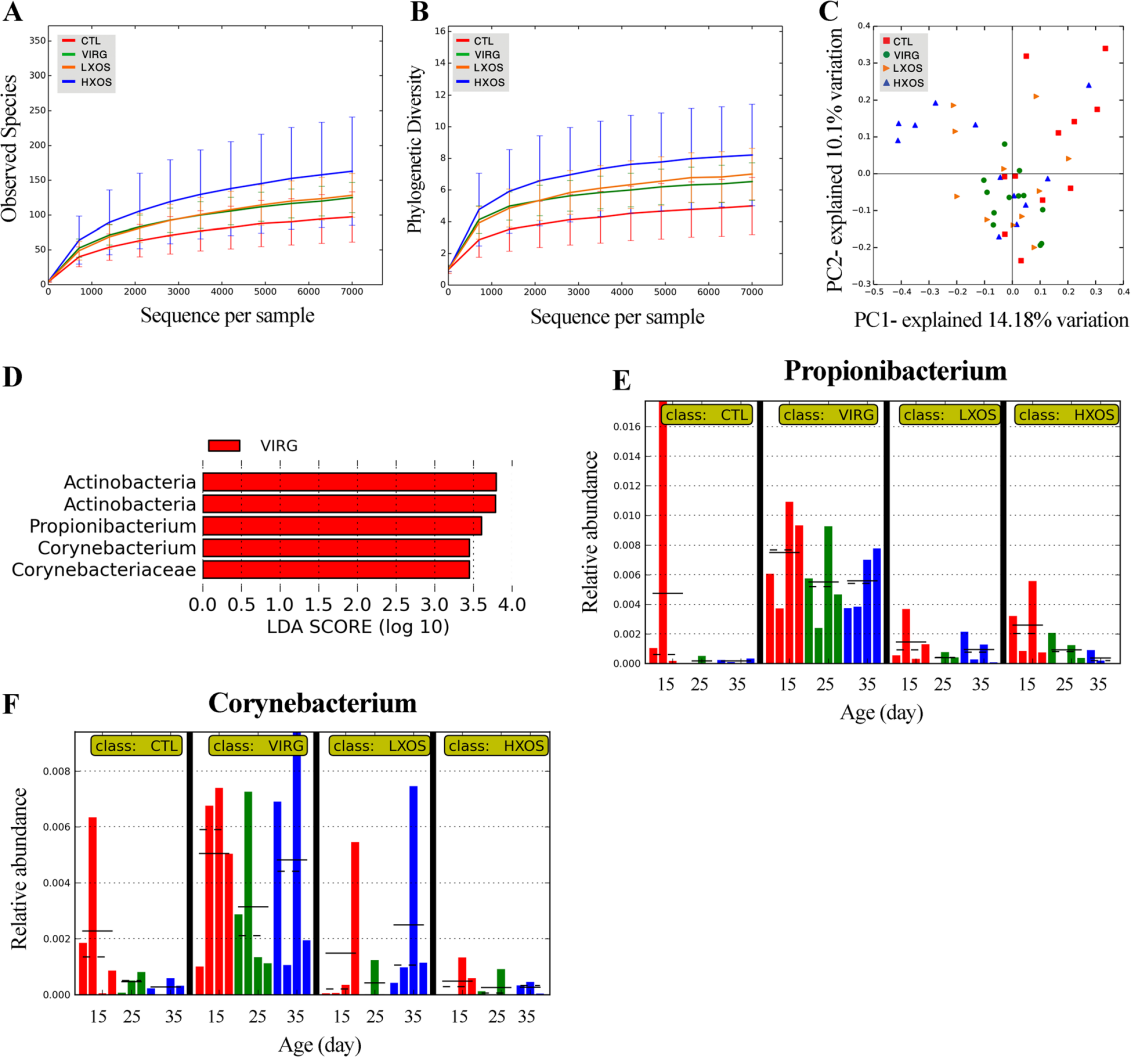
**Treatment effects on ileal microbiota diversity and composition.** Rarefaction curves, calculated at the lowest subsample size of 7,000 sequences per sample, show the effects of sequencing efforts on the observed number of OTUs at 97% sequence similarity **(A)** and phylogenetic diversity (whole tree) **(B)**. Data are calculated at 3% distance. Error bars show standard deviation for each category. Principal component analysis (PCoA) of unweighted UniFrac distances from 24 ileal samples shows no difference in the community phylotype structure among treatments **(C)**. Linear discriminant analysis (LDA) effect size (LEfSe) was used to identify specific phylotypes that significantly associated with treatments. LDA more than 2 reflects significant difference between groups. LEfSe analysis provided the list of phylotypes that are differential among dietary supplementations with statistical and biological significance **(D)**. The histograms indicated the increased relative abundance of the genera *Propionibacterium* **(E)** and *Corynebacterium* **(F)** in the ileal microbiota of chickens fed VIRG diet compared with other treatments. Each bar represents the relative abundance of the taxa in a sample at the age of 15 (red line), 25 (green line), and 35 days (blue line). The mean and median relative abundance are indicated with solid and dashed lines, respectively. CTL: control diet without any antibiotic or prebiotic; VIRG: control diet supplemented with 16.5 mg virginiamycin; LXOS: control diet supplemented with 1 g/kg xylo-oligosaccharides; HXOS: control diet supplemented with 2 g/kg xylo-oligosaccharides.

To assess the within-community (α) diversity, the number of observed OTUs (at the 97% level) and phylogenetic diversity (PD) were calculated. None of the dietary treatments had a significant effect on the α-diversity indices of the ileal bacterial community (*P* > 0.05; Figure 1A,B and Additional file 1: Table S1). Rarefaction curves for the observed OTUs (Figure 1A) and PD values (Figure 1B) approach a plateau, indicating that sequencing depth was sufficient for the coverage of all OTUs present in ileal samples. Although the number of observed OTUs and the PD values were higher in chickens fed HXOS, these differences did not reach a statistical significance (*P* > 0.05; Additional file 1: Table S1). PD values differed most between the HXOS and control group (*P* = 0.06). To determine similarities between pairs of microbial communities (β-diversity), a principal component analysis (PCoA) was performed using unweighted UniFrac distance matrices. Because of high inter-individual variation, no distinguishable clustering of the samples was evident based on the dietary treatments (Analysis of similarities (ANOSIM): *R* = 0.09, *P* = 0.006; Figure 1C). These results demonstrated that chickens shared a core set of microbiota in the ileum regardless of dietary supplementation.

OTUs were taxonomically assigned with the Ribosomal Database Project (RDP) classifier at 80% confidence threshold. The relative abundance of OTUs was analyzed at different ranking levels from phylum to genus. At the phylum level, ileal microbiota was mainly composed of *Firmicutes* (>85%) followed by *Proteobacteria*, *Actinobacteria*, and *Bacteroidetes* (Additional file 1: Figure S1). We used the linear discriminant analysis (LDA) effect size (LEfSe) method and identified five taxonomic biomarkers (LDA >2) in the ileal microbial community of VIRG-treated birds (Figure 1D). The relative abundance of two genera, *Propionibacterium* (Figure 1E) and *Corynebacterium* (Figure 1F) in *Actinobacteria* phylum was significantly (LDA >2) higher in the VIRG group compared with other dietary groups.

### Effects of dietary treatments on the cecal microbiota

**Figure 2:**
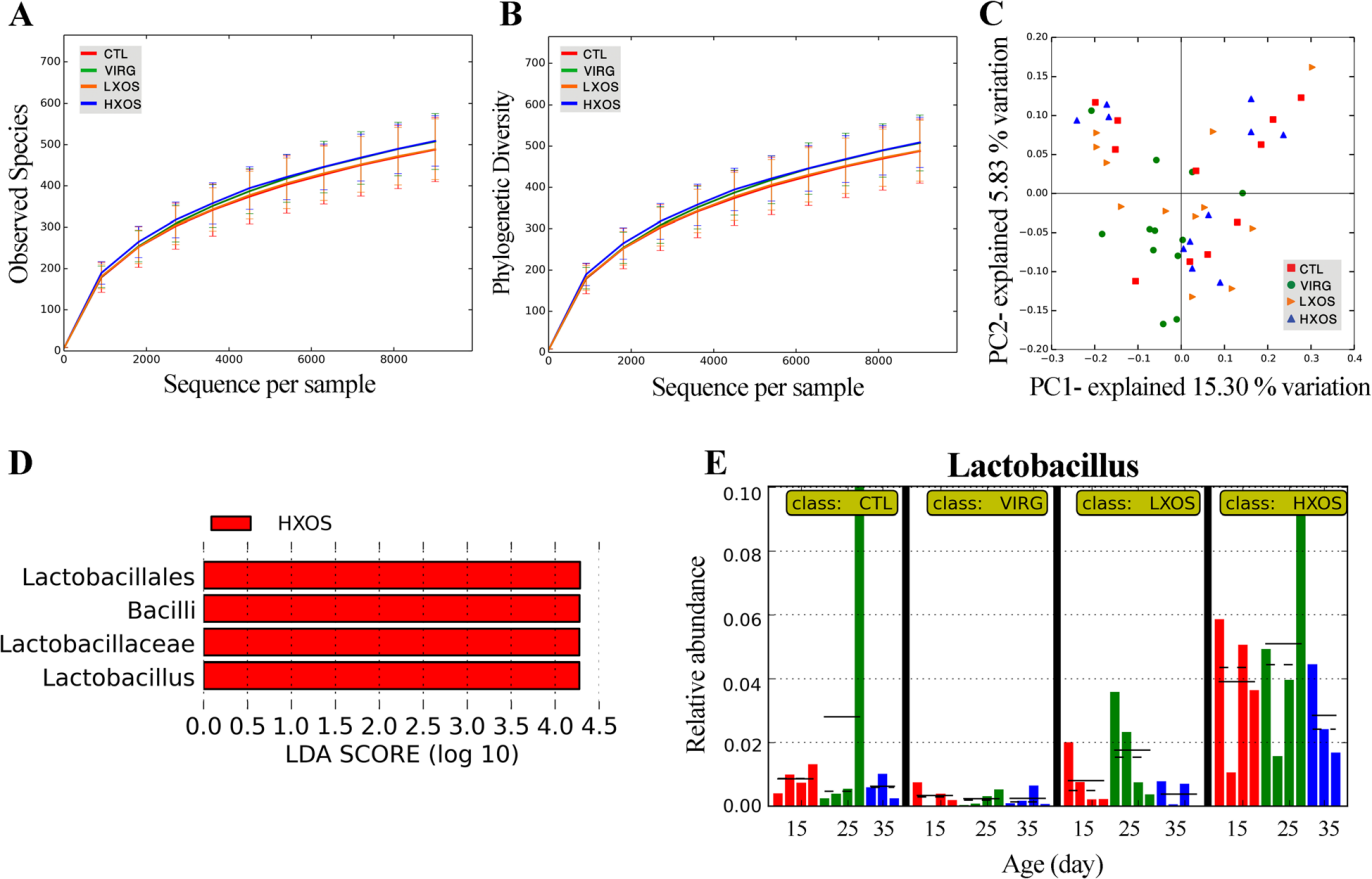
**Treatment effects on diversity and composition of cecal microbiota.** Alpha diversity indices were calculated on rarefied samples at the lowest subsample size of 9,000 sequences per sample. There was no significant (nonparametric *t*-test, *P* > 0.05) effects of prebiotic or antibiotic on the observed number of OTUs **(A)** and phylogenetic diversity (whole tree) **(B)**. Error bars show standard deviation for each category. Unweighted UniFrac PCoA plot shows no separation of bacterial communities between dietary groups **(C)**. Key phylotypes of cecal microbiota responding to dietary treatments were identified using LEfSe algorithm **(D)**. The histogram shows the increased abundance of the genus *Lactobacillus* in the cecal microbiota of chickens fed HXOS diet compared with other treatments **(E)**. Each bar represents the relative abundance of the taxa in a sample at the age of 15 (red line), 25 (green line), and 35 days (blue line). The mean and median relative abundance are indicated with solid and dashed lines, respectively. CTL: control diet without any antibiotic or prebiotic; VIRG: control diet supplemented with 16.5 g/ton virginiamycin; LXOS: control diet supplemented with 1 g/kg xylo-oligosaccharides; HXOS: control diet supplemented with 2 g/kg xylo-oligosaccharides.

Rarefaction curves of 9,000 subsampled reads in the cecum showed comparable numbers of OTUs (at the 97% identity level) for each dietary treatment (Figure 2A and Additional file 1: Table S1). Similarly, there was no apparent difference (*P* > 0.05) in rarefaction curves for the PD values (Figure 2B and Additional file 1: Table S1). The microbial community structure between dietary treatments (β-diversity) was compared using PCoA of the unweighted UniFrac distances. These PCoA plots showed that microbial communities from XOS- and VIRG-supplemented birds did not clearly separate from those of the non-supplemented birds (*R* = 0.02, *P* = 0.78). The first axis of the PCoA explained 15.3% of the variation in bacterial diversity while the second axis explained 5.8% (Figure 2C). More than 99% of the sequences were assigned to bacterial phyla with the RDP classifier. At the phylum level, the cecal microbiota was dominated by *Firmicutes* (>80%), followed by *Proteobacteria* and *Bacteroidetes* (Additional file 1: Figure S1). LEfSe detected a marked increase (LDA score >4) in the relative abundance of the *Lactobacillus* genus in chickens fed HXOS compared to other treatment groups (Figure 2D). Figure 2E shows the histogram of the relative abundance of *Lactobacillus* in each treatment at different time points.

### Comparison between the ileal and the cecal microbiota

**Figure 3:**
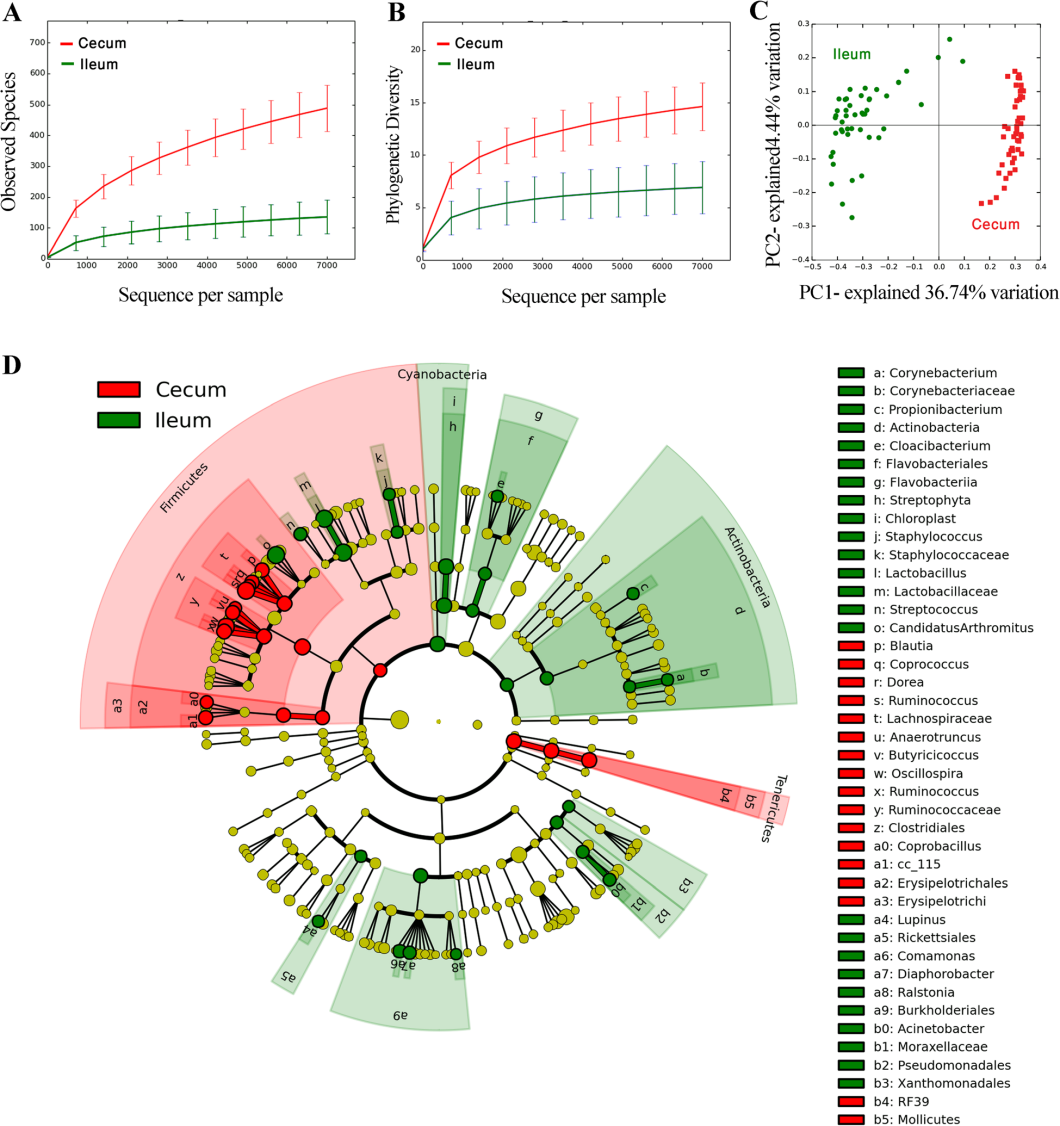
**Differences between ileal and cecal microbiota of broiler chickens.** Rarefaction curves for ileal and cecal bacteria, for the observed OTUs **(A)** and phylogenetic diversity **(B)** indices. Significant differences were seen between the alpha diversity indices of ileal samples and samples taken from cecum (nonparametric *t*-test, *P* < 0.01). PCoA analysis of OTUs **(C)** indicates that the bacterial profile differed strongly according to sampling site (*R* = 094, *P* = 0.001). Taxa significantly associated with ileal communities (green) *versus* cecal communities (red), shown in circular cladogram based on the Ribosomal Database Project (RDP) bacterial taxonomy **(D)**. The cutoff value of linear discriminant analysis (LDA) was 2.0 or higher. Biomarker taxa are heighted by colored circles and shaded areas. Each circle’s diameter is relative to abundance of taxa in community.

The number of observed OTUs and PD values were higher (*P* < 0.001) in the cecal samples than those in corresponding ileal samples (Figure 3A,B), indicating that the cecal microbiota was more diversified than the ileal microbiota. The PCoA of OTUs from the ileum and cecum (Figure 3C) also demonstrated that the bacterial community structure differed significantly according to sampling site (*R* = 0.94, *P* = 0.001). In addition, the phylogenetic composition of the microbiota was noticeably different between ileum and cecum samples. LEfSe results showed that 46 bacterial clades at all taxonomic levels were differentially abundant (LDA score >2.0) between the ileal and cecal microbiota (Figure 3D). *Lactobacillaceae* and *Clostridiaceae* were the dominant families in the ileum while the cecum was inhabited mostly by the *Lachnospiraceae* and *Ruminococcaceae* families (Additional file 1: Figure S1). The most dominant genera in the cecum were *Ruminococcus* and *Oscillospira* accounting for greater than 35% of all observed sequences.

### Temporal changes in the ileal and the cecal microbiota

**Figure 4:**
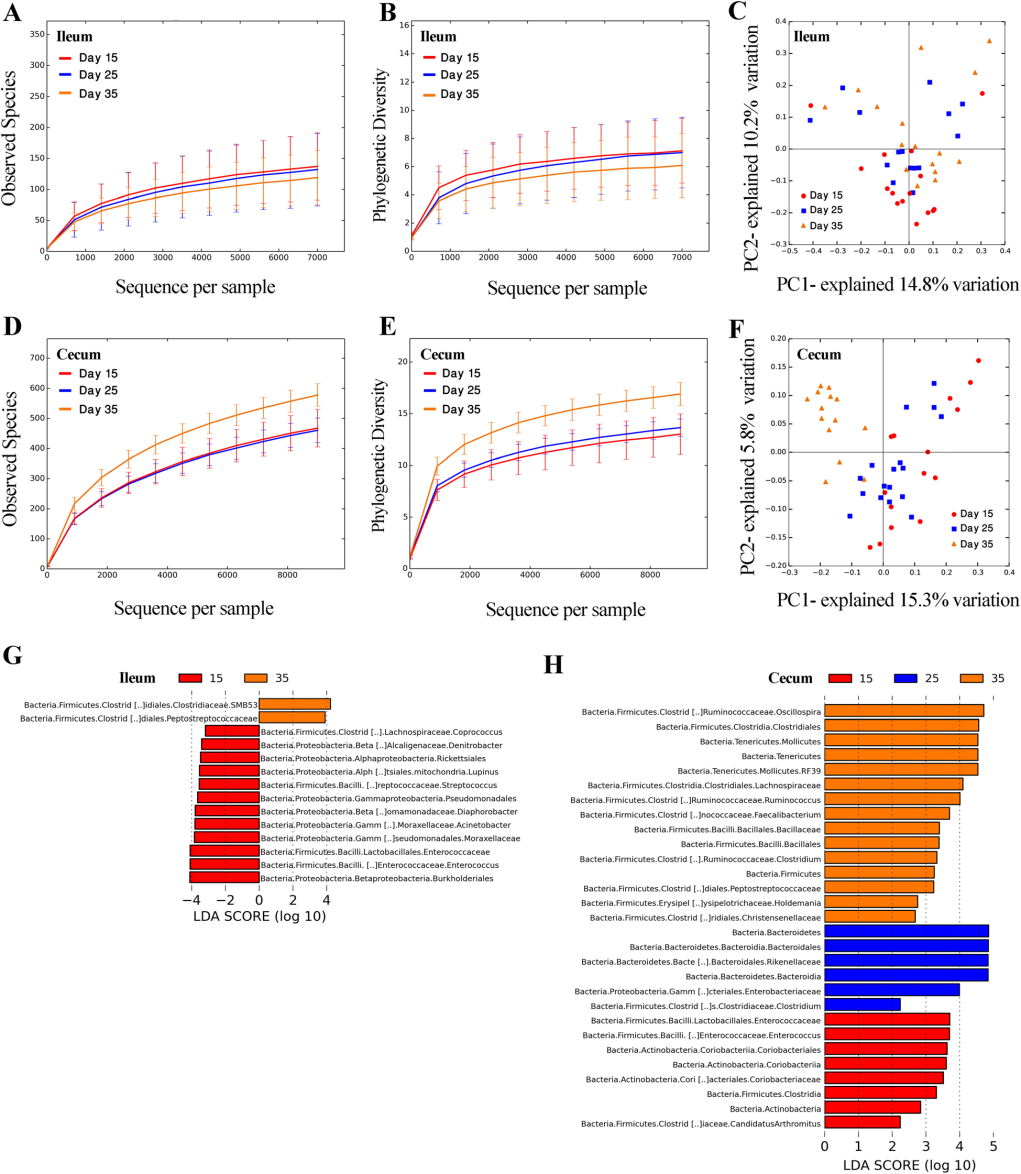
**Ileal and cecal microbiota of broiler chickens at different ages.** Observed OTUs **(A)** and phylogenetic diversity **(B)** rarefactions of ileal samples collected on days 15, 25, and 35. Unweighted Unifrac PCoA shows a statistically, but not biologically, significant effect of age on the ileal microbiota (*R* = 0.1, *P* = 0.01) **(C)**. Alpha rarefaction analysis of cecal samples (**D**, **E**) shows that observed OTUs and phylogenetic diversity both increased on day 35 compared with those samples collected on day 15 and 25 (*P* < 0.01). A separate clustering of cecal microbiota was observed on day 35 (*R* = 0.42, *P* < 0.01) **(F)**. The LEfSe provided the list differentially abundant taxa between ages in the ileum **(G)** and cecum **(H)**. The cutoff value of linear discriminant analysis (LDA) was 2.0 or higher.

To determine whether the age of the birds affected the gut microbiota, the ileal and cecal microbiota of chickens at different ages, 15, 25, and 35 days old, in each treatment group were compared. Rarefaction plots indicated no significant (*P* > 0.05) changes in α-diversity metrics of the ileal samples at three different time points (Figure 4A,B and Additional file 1: Table S2). Unweighted Unifrac PCoA revealed the statistical significant effect of age on the ileal samples (*P* = 0.01) but the *R* value was relatively small (*R* = 0.1) and therefore the difference was probably not biologically significant (Figure 4C). In the cecum, a marked increase (*P* < 0.01) in observed OTUs and the PD values occurred on day 35 compared with cecal samples from day 15 and 25 (Figure 4D,E and Additional file 1: Table S2). The PCoA plot of unweighted UniFrac distances indicated a clear separation between samples from day 35 and samples taken at days 15 and 25 (*R* = 0.42, *P* < 0.01; Figure 4F). Bacteria that were differentially abundant between sampling times in the ileum and cecum were detected using LEfSe (Figure 4G,H). For example, in the ileum, the order *Burkholderiales* and the candidate genus SMB53 of *Clostridiaceae* were the most differentially abundant taxa at days 15 and 35, respectively (Figure 4G). The most differentially overrepresented taxa in the cecum at 15, 25, and 35 days of age were the genus *Enterococcus*, family *Rikenellaceae* and genus *Oscillospira*, respectively (Figure 4H).

### SCFA in the ileum and cecum

**Figure 5:**
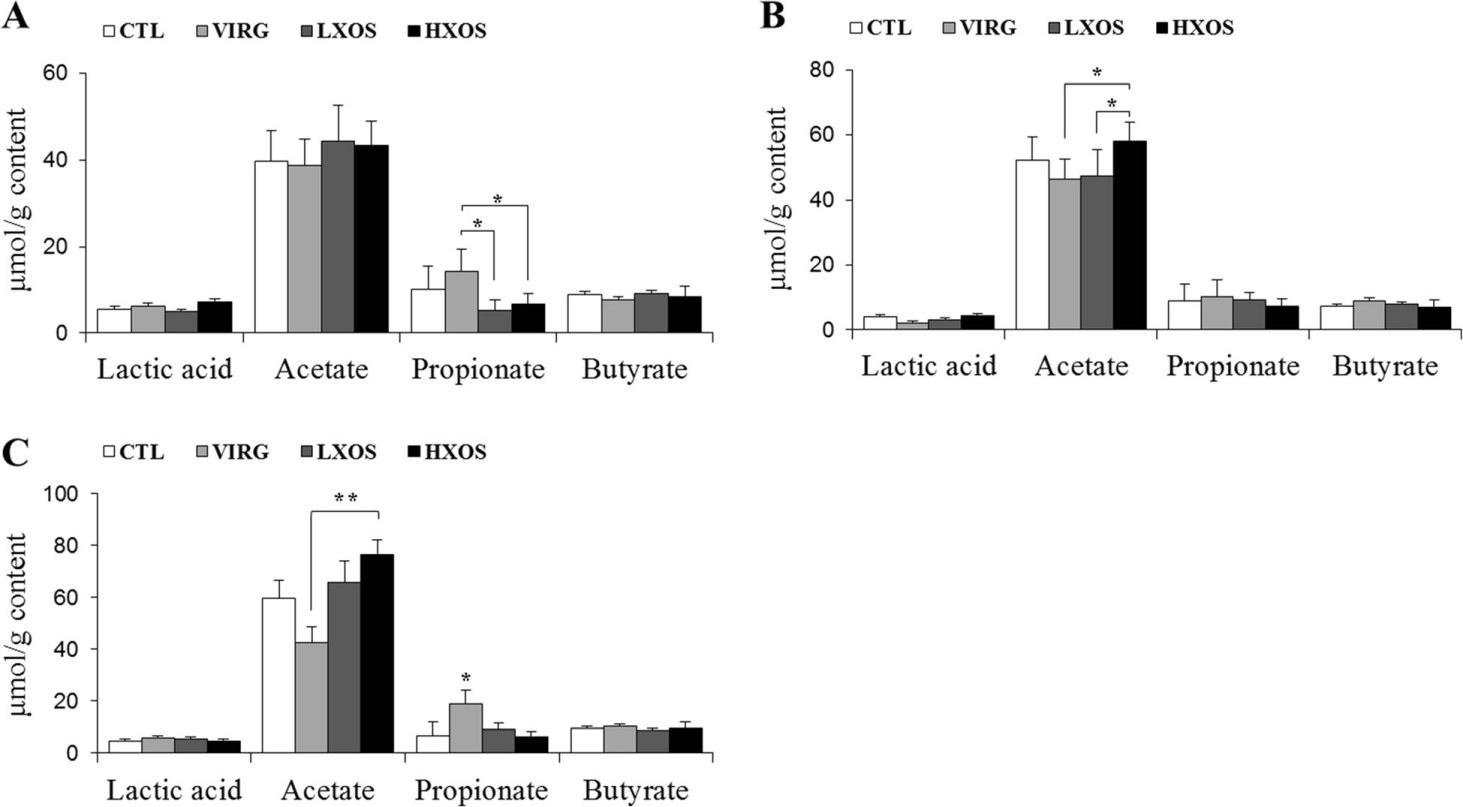
**SCFA and lactate concentrations in the cecum.** Lactate, acetate, propionate, and butyrate concentrations (μmol/g content) of 24 cecal samples collected on 15 **(A)**, 25 **(B)**, and 35 **(C)** days of age. Significant differences (*P < 0.05 and **P < 0.01) were detected using the Scheffe’s multiple comparison test by ANOVA. CTL: control diet without any antibiotic or prebiotic; VIRG: control diet supplemented with 16.5 g/ton virginiamycin; LXOS: control diet supplemented with 1 g xylo-oligosaccharides/kg; HXOS: control diet supplemented with 2 g xylo-oligosaccharides/kg.

To further identify whether the observed microbial changes due to dietary treatment also affected the gut function, SCFA and lactate concentrations were measured. In the ileum, the acetate concentration ranged from 6.2 to 7.1 μmol/g digesta and was not affected by dietary treatments, while lactate, propionate and butyrate were not detected (data not shown). The cecal propionate concentration in the VIRG group was significantly higher (*P* < 0.05) than LXOS and HXOS groups on day 15 (Figure 5A) and also significantly higher than the control, LXOS and HXOS groups on day 35 (Figure 5C). The cecal acetate concentration was significantly higher in the HXOS group than in the VIRG group on days 25 and 35 (*P* < 0.05; Figure 5B,C) and the LXOS group on day 25 (*P* < 0.01; Figure 5B). Correlation analyses showed that the relative abundance of the *Lactobacillus* genus in the cecum was positively correlated with cecal acetate production (*R* = 0.57, *P* < 0.05), whereas ileal *Propionibacterium* relative abundance was positively correlated with cecal propionate concentrations (*R* = 0.51, *P* < 0.05).

### Growth performance

Results for production traits of broilers through the experimental period are shown in Table 1. The average body weight of chickens did not differ among treatments. The feed conversion ratio (FCR) in broilers fed VIRG and HXOS diets was lower (*P* < 0.05) than those fed CTL or LXOS between days 7 and 21. No difference was observed in FCR during the first week and last 15 days of experiment. The mortality rate of chickens was not affected by dietary treatments and was lower than 7% in all groups.

**Table 1:** **Growth performance of broilers in different treatment groups**

**Parameter**	**Treatments**				**SEM**
	**CTL**	**VIRG**	**LXOS**	**HXOS**	
Body weight (g)					
Day 0	38.9	38.5	40.0	39.6	4.2
Day 7	261.5	254.4	269.7	259.0	18.3
Day 21	733.6	753.0	728.2	741.5	29.1
Day 35	1,892.0	1,909.0	1,891.2	1,914.7	26.7
Feed conversion ratio					
Days 0 to 7	0.95	0.97	1.00	1.02	0.081
Days 7 to 21	1.77^a^	1.61^b^	1.75^a^	1.62^b^	0.044
Days 21 to 35	2.14	2.09	2.17	2.13	0.078
Survival (%)	93.3	93.3	96.6	93.3	1.0

CTL: control diet without any antibiotic or prebiotic; VIRG: control diet supplemented with 16.5 g/ton virginiamycin; LXOS: control diet supplemented with 1 g/kg xylo-oligosaccharides; HXOS: control diet supplemented with 2 g/kg xylo-oligosaccharides; SEM: standard error of the mean. Each mean represents six replicate cages with five broilers per cage. ^a,b^Means in the same row with different superscripts differ (*P* < 0.05).

## Discussion

High-throughput sequencing of 16S rRNA gene amplicons has been used more recently to identify functional diversity [20] or variability [21] of the microbiome in the gut of broiler chickens. However, on the subject of dietary supplementation with XOS or VIRG, previous studies have used either low-resolution bacterial detection techniques [16-18] or considered an antibiotic mixture, rather than VIRG alone [19]. In the present study, we used high-throughput sequencing of the V1-V3 region of the 16S rRNA gene to monitor the ileal and cecal microbiota of a large number of individual chickens fed either a sub-therapeutic level of VIRG or one of two levels of XOS over a 5-week production cycle. VIRG is one of the most commonly used in-feed antibiotics in the poultry industry for disease prevention and growth promotion. Our results indicate that VIRG and XOS differentially modified the proportion of specific OTUs and these changes were associated with cecal acetate and propionate production.

Based on the phylogenetic diversity of bacterial communities and number of observed OTUs, we concluded that the VIRG inclusion (16.5 g/ton) did not change the chicken ileal and cecal bacterial community membership. Similar results have been previously reported in chicken [19,22] and swine gut microbiota following treatments with in-feed antibiotics [23-25]. However, VIRG treatment significantly altered relative abundance of certain taxa in the ileum whereas no effect was observed on the cecal microbial composition. This observation is in accordance with the study of Dumonceaux *et al*. [18] who reported that virginiamycin addition (20 g/ton) altered the chicken gut microbiota most significantly in the upper intestinal tract. In contrast, Danzeisen *et al*. [19] described a number of changes in the proportion of taxa including a reduction in lactobacilli and an increase in *Escherichia coli* in the cecal contents of chicken exposed to a mixture of monensin (110 g/ton) with virginiamycin (15 g/ton) or tylosin (20 g/ton). This discrepancy is likely due to the higher dose of antibiotic used in their study. Interestingly, lactobacilli was not identified as a biomarker of VIRG treatment in our study, although it is generally considered to be reduced with antibiotics [17,19,26]. The relatively low levels of antibiotics used in Canadian poultry industry and in the present study may be responsible for the lack of significant changes in the gut bacterial community membership or the cecal microbial composition in the current study.

We identified two genera of bacteria as being linked to VIRG treatment, namely *Corynebacterium* and *Propionibacterium*. Dumonceaux *et al*. [18] also observed an increase in *Corynebacterium glutamicum* in the proximal intestinal of virginiamycin-treated chickens, using quantitative PCR method. Under anaerobic conditions, *C. glutamicum* catabolizes different carbohydrates and produces organic acids such as lactate and succinate [27]. The genus *Propionibacterium* is a gram-positive bacterium with a unique ability to produce propionate. In our study, the propionate concentration was lower than the limit of detection in the ileum. However, a marked increase in cecal propionate concentration was observed as a result of the VIRG treatment and was positively correlated with the change in the relative abundance of *Propionibacterium* in the ileum. The immunomodulatory effects of selected strains of *Propionibacterium* such as *Propionibacterium freudenreichii* and *Propionibacterium acidipropionici* have been established in humans and animals [28-31]. *Propionibacterium* species are also able to bind to aflatoxin B1 and reduce its intestinal absorption in chickens [32]. Aflatoxin B1 is a major food contaminant in poultry production that depresses growth performance. At this point, it is unclear whether the improved feed efficiency in VIRG-fed chickens is related to the increased relative abundance of *Corynebacterium* and *Propionibacterium* or not. We hypothesize that these genera may contribute to reported growth promoting functions of antibiotic.

While XOS are not digestible by gastrointestinal digestive enzymes, they can be fermented by the gut microbiota, producing SCFA and lactate [33]. Previous studies on humans [34], rats [35,36], and chickens [16,37] have analyzed cultivable members of the fecal and cecal microbiota and found that XOS is effective in promoting intestinal health by encouraging the growth of beneficial bacterial species. Our results demonstrate that the cecal microbiota of HXOS-fed chickens contained significantly higher proportions of the genus *Lactobacillus* than the other dietary treatments. Several strains of *Lactobacillus* have been identified as functional probiotics with associated anti-inflammatory and antimicrobial activities. *In vitro* fermentation of XOS by *Lactobacillus brevis* and *Lactobacillus fermentum* has been previously reported [11]. Lactate produced by *Lactobacillus* species, is rarely accumulated and is mostly converted to butyrate and acetate as shown by *in vitro* studies [38,39]. This was substantiated in the current study by the increased production of acetate after HXOS supplementation in comparison with the VIRG and LXOS groups.

The average number of high-quality sequencing reads obtained per sample in this study was higher than previously published studies of the chicken gut microbiota [19-21]. This has enabled us to provide a more comprehensive view about the ileal and cecal microbiota composition and structure. The ileal microbiota was mainly composed of *Firmicutes* (>85%), and within this phylum, the majority belonged to the *Lactobacillus* genus, a finding that is consistent with previous 16S rRNA gene-based studies [40,41]. In addition to *Lactobacillus*, an unknown genus in the *Clostridiaceae* family was reported to be dominant in the ileum [41]. This was identified as the genus *Candidatus Arthromitus* in our study. In cecal samples, most of the OTUs were classified as, *Oscillospira, Ruminococcus* and unknown genera of the *Lachnospiraceae* and *Clostridiaceae* families, which was in accordance with the earlier pyrosequencing-based studies [19,42].

Furthermore, we found that cecal bacterial diversity increased over time, similar to what has been previously observed in chicken [19,22,43] and wild bird species [44]. In addition, the relative abundance of certain bacterial families or genera was altered over time in the ileum and cecum of the chickens. For example, the relative abundance of the genus *Enterococcus* declined whereas *Faecalibacterium* and *Clostridium* increased in the cecum with increasing age. It was also noted that *Enterobacteriaceae*, a family that comprises many known pathogens such as *Salmonella*, *Shigella*, and *E. coli*, was more abundant in the ileum and cecum of the young birds. Similar results were obtained by Wise *et al*. [45] in a quantitative PCR-based study using group-specific 16S rDNA primers. They reported that members of *Enterobacteriaceae* are the most abundant in the cecum at day 7 but being replaced by obligate anaerobe sequences by days 14 and 21. The ileal bacterial community appeared to be more stable than the cecum, a finding that was similar to the observation of Lu *et al*. [46]. Bird age, as evident from this study and others [19,22], had a higher impact on gut microbiota as compared to dietary treatments.

## Conclusions

Taken together, this study indicates that dietary prebiotic or sub-therapeutic antibiotic supplementation modulated the relative abundance of specific bacteria without changing the overall microbial structure. We showed that bacterial community clustering was mainly due to the sample location and the age of the birds rather than dietary supplementation. Increased population levels of lactate-producing bacteria and elevated cecal acetate concentrations in chickens fed HXOS might be an intestinal health-promoting attribute and may contribute to improved feed efficiency during the growth period.

## Methods

### Birds, diet, and experimental design

One hundred and twenty male 1-day-old broiler chickens (Ross × Ross) were obtained from a local commercial hatchery and grown over a 35-day experimental period at the Macdonald Campus Poultry Complex, McGill University. Birds were randomly assigned to one of four dietary treatments (six cage replicates; five birds/cage) which included 1) CTL: a commercial and typical broiler diet without any supplements, 2) VIRG: diet 1 supplemented with sub-therapeutic levels of virginiamycin (16.5 g/ton diet), 3) LXOS: diet 1 + 1 g XOS/kg, and 4) HXOS: diet 1 + 2 g XOS/kg. The main ingredients of the diets were corn and soybean meal, formulated according to the NRC requirement (Additional file 1: Table S3). Chickens had free access to feed and water. Birds were raised under controlled environmental conditions with an 18-h lighting cycle and a temperature of 32°C at day 1 which was gradually reduced and maintained at 24°C on day 10. Body weight (BW) and feed intake were recorded on a cage-by-cage basis on days 7, 21, and 35. FCR was calculated as feed intake (kg) divided by body mass gain (kg). The animal use protocol was according to the guidelines of the Canadian Council on Animal Care and approved by the Animal Ethics Committee of McGill University (Protocol no. 2012–6073).

### Sample collection and DNA extraction

Four birds per treatment were randomly chosen at three different time points, 15, 25, and 35 days of age, and euthanized by electrical stunning and carotid artery bleeding. The ileum (about 2 cm proximal to cecal tonsils) and cecum were collected within 5 min of euthanasia, immediately placed in cryogenic vials, snap-frozen in liquid nitrogen, delivered to the laboratory and stored at −80°C until DNA extraction. Total genomic DNA was isolated from 220 mg of frozen ileal and cecal contents using the QIAamp DNA Stool Mini Kit (Qiagen, Toronto, ON, Canada). The DNA concentration and purity was determined using a NanoDrop 2000 spectrophotometer (Thermo Scientific, MA, USA).

### Pyrosequencing

The normalized concentration (20 ng/μl) of purified genomic DNA was used as a template to analyze the microbial communities. The V1-V3 region of the 16S rRNA gene was amplified using universal eubacterial primers (27 F: AGRGTTTGATCMTGGCTCAG and 519R: GTNTTACNGCGGCKGCTG) [47]. Unique 8 nucleotide sample-specific barcodes and Roche 454 A-adapters (Roche, Basel, Switzerland) were fused to the 5′ end of the forward primer while the B-adapters were added to the 5′ end of the reverse primer. PCR reactions were performed by initial denaturation at 94°C for 3 min and then 28 cycles of 94°C for 30 s, 53°C for 40 s and 72°C for 1 min, followed by a final elongation step at 72°C for 5 min. PCR products were purified using the MinElute kit (Qiagen, Toronto, ON, Canada). Amplicon pyrosequencing was performed at the MR DNA sequencing center (Shallowater, TX, USA) using 454 GS FLX technology.

### Data processing

Sequence reads were analyzed by the quantitative insights into microbial ecology (QIIME) v.1.8.0 software package [48]. Briefly, sequences were demultiplexed and assigned to individual samples according to the specific barcode of each sample. Barcodes and primers were trimmed, where maximum two base differences in barcodes and no primer mismatches were permitted. Sequences were excluded if they were not meeting the default QIIME quality criteria. Sequences with an average quality score less than 25 in a sliding window of 50 nucleotides were also discarded. The sequence data were denoised using the *denoise_wrapper.py* command [49] within QIIME. The chimeras were identified using the UCHIME method [50] against the GOLD database and removed from further analyses.

The remaining quality-filtered reads were clustered *de novo* (97% similarity threshold) into OTUs using the CD-HIT method [51], and the most abundant sequence was selected as the OTU representative. The sequence alignment was performed against the Greengenes core set using the PyNAST method [52]. OTUs were taxonomically categorized using the naïve Bayesian RDP classifier [53] trained on the Greengenes database with a minimum confidence score of 0.8. For downstream analysis, the OTU table was filtered by discarding OTUs that comprised fewer than 0.005% of all sequences [54].

### Cecal SCFA and lactate concentrations

For determination of cecal SCFA and lactate concentrations, 0.5 g of fresh cecal contents were diluted in 1 ml of 10% perchloric acid, homogenized, and centrifuged at 15,000 × *g* for 10 min at 4°C. The supernatant was filtered through syringe filters with 25-mm diameter membrane and stored at −20°C. The samples (20 μl) were injected into a high performance liquid chromatograph (HPLC) system equipped with a Varian ProStar AutoSampler (Hamilton, Reno, NV, USA), a UV detector (210 nm), and an ion-exclusion Aminex HPX-87H 300 × 7.8 mm column (Bio-Rad, Hercules, CA, USA). The column was maintained at room temperature with 0.013 M sulfuric acid as the eluent (0.6 ml/min flow rate). Lactate, acetate, propionate, and butyrate in the samples were quantified using external calibration curves.

### Statistical measurements

To compare the microbial community structure, unweighted UniFrac distance matrices were computed using the OTU table and phylogenetic tree information to serve as input to plot PCoA using QIIME. ANOSIM with 999 permutations was used to detect statistical significances between microbial communities in different groups. This test measures a value of *R*, normally scaled from 0 to 1, which is based on the average rank similarity among groups and replicates within each group [55]. *R* = 0 indicates that two groups are similar whereas *R* = 1 shows a perfect separation between groups. Differentially abundant taxa were identified using the LEfSe method [56]. The LEfSe algorithm uses the nonparametric factorial Kruskal-Wallis test (α = 0.05) to analyze differences between classes (treatments) and the pairwise Wilcoxon test (α = 0.05) to check differences among subclasses (time points) within different classes. To evaluate the α-diversity in samples, the rarefaction curves of PD and number of observed OTUs were computed using QIIME. To normalize the sequencing depth, the lowest counts among samples were randomly subsampled in each library 1,000 times and average values were used to measure diversity indices. The differences between the mean values were identified by analyses of variance (ANOVA) and Scheffe’s multiple comparison test using SAS v9.1 software. PROC CORR was used to analyze the Pearson correlation between bacterial genera and SCFA concentrations. The differences of growth performance parameters among treatments were analyzed by one-way ANOVA, and each cage was considered as an experimental unit.

## Availability of supporting data

The raw sequence data obtained in this study has been deposited in the Sequence Read Archive (SRA) database of the National Center for Biotechnology Information (NCBI) with an access number of SRP044612.

## Additional file

Additional file 1:
**Supplementary data.** **Table S1.** Statistical comparison of alpha diversities between treatments in the ileal and cecal microbiota. A nonparametric *t*-test was run in QIIME to compare the alpha diversities using the default number of Monte Carlo permutations (999) and the greatest rarefaction depth. Cells shaded with light blue (upper right section) shows the *P* values for the number of observed species while those shaded with light yellow (lower left section) shows the *P* values of phylogenetic diversity comparison. **Table S2.** Statistical comparison of alpha diversities between ages in the ilea and cecal microbiota. A nonparametric two sample *t*-test was run in QIIME to compare the alpha diversities using the default number of Monte Carlo permutations (999) and the greatest rarefaction depth. Cells shaded with light blue (upper right section) shows the *P* values for the number of observed species while those shaded with light yellow (lower left section) shows the *P* values of phylogenetic diversity comparison. Significant *P* values are bolded. **Table S3.** Ingredient composition (g/kg) of experimental diets. **Figure S1.** The ileal and cecal microbial composition on days 15, 25, and 35. The bar charts indicate the relative abundance (%) of bacterial phyla (A) and the dominant (>0.5% of sequences) bacterial families and genera (B) in the ileum and cecal microbiota of chickens. Classification is according to the RDP trained on the Greengenes database with a minimum confidence score of 0.8. For each habitant, ileal and cecal contents of 16 birds were collected at 15, 25, and 35 days old.

## Abbreviations

AGP: antibiotic growth promoter
FCR: feed conversion ratio
LDA: linear discriminant analysis
LEfSe: linear discriminant analysis effect size
OUT: operational taxonomic unit
PCoA: principal component analysis
PD: phylogenetic diversity
RDP: Ribosomal Database Project
SCFA: short chain fatty acids
STD: standard deviation
VIRG: virginiamycin
XOS: xylo-oligosaccharides

## References

[1] Castanon J (2007). History of the use of antibiotic as growth promoters in European poultry feeds. Poult Sci.

[2] Maron DF, Smith TJ, Nachman KE (2013). Restrictions on antimicrobial use in food animal production: an international regulatory and economic survey. Global Health.

[3] Casewell M, Friis C, Marco E, McMullin P, Phillips I (2003). The European ban on growth-promoting antibiotics and emerging consequences for human and animal health. J Antimicrob Chemother.

[4] Dibner J, Richards J (2005). Antibiotic growth promoters in agriculture: history and mode of action. Poult Sci.

[5] Oakley BB, Lillehoj HS, Kogut MH, Kim WK, Maurer JJ, Pedroso A (2014). The chicken gastrointestinal microbiome. FEMS Microbiol Lett.

[6] Smith PM, Howitt MR, Panikov N, Michaud M, Gallini CA, Bohlooly-Y M (2013). The microbial metabolites, short-chain fatty acids, regulate colonic Treg cell homeostasis. Science.

[7] Arpaia N, Campbell C, Fan X, Dikiy S, van der Veeken J, Liu H (2013). Metabolites produced by commensal bacteria promote peripheral regulatory T-cell generation. Nature.

[8] Pourabedin M, Xu Z, Baurhoo B, Chevaux E, Zhao X (2014). Effects of mannan oligosaccharide and virginiamycin on the cecal microbial community and intestinal morphology of chickens raised under suboptimal conditions. Can J Microbiol.

[9] Aachary AA, Prapulla SG (2011). Xylooligosaccharides (XOS) as an emerging prebiotic: microbial synthesis, utilization, structural characterization, bioactive properties, and applications. Compr Rev Food Sci Food Saf.

[10] Broekaert WF, Courtin CM, Verbeke K, Van de Wiele T, Verstraete W, Delcour JA (2011). Prebiotic and other health-related effects of cereal-derived arabinoxylans, arabinoxylan-oligosaccharides, and xylooligosaccharides. Crit Rev Food Sci Nutr.

[11] Moura P, Barata R, Carvalheiro F, Gírio F, Loureiro-Dias MC, Esteves MP (2007). *In vitro* fermentation of xylo-oligosaccharides from corn cobs autohydrolysis by *Bifidobacterium* and *Lactobacillus* strains. LWT-Food Science and Technology.

[12] Kondepudi KK, Ambalam P, Nilsson I, Wadström T, Ljungh Å (2012). Prebiotic-non-digestible oligosaccharides preference of probiotic bifidobacteria and antimicrobial activity against *Clostridium difficile*. Anaerobe.

[13] Gullón P, Moura P, Esteves MAP, Girio FM, Domínguez H, Parajó JC (2008). Assessment on the fermentability of xylooligosaccharides from rice husks by probiotic bacteria. J Agric Food Chem.

[14] Madhukumar M, Muralikrishna G (2012). Fermentation of xylo-oligosaccharides obtained from wheat bran and Bengal gram husk by lactic acid bacteria and bifidobacteria. J Food Sci Technol.

[15] Mäkeläinen H, Saarinen M, Stowell J, Rautonen N, Ouwehand A (2010). Xylo-oligosaccharides and lactitol promote the growth of Bifidobacterium lactis and Lactobacillus species in pure cultures. Benefic Microbes.

[16] Courtin CM, Swennen K, Broekaert WF, Swennen Q, Buyse J, Decuypere E (2008). Effects of dietary inclusion of xylooligosaccharides, arabinoxylooligoscaccharides and soluble arabinoxylan on the microbial composition of caecal contents of chickens. J Sci Food Agric.

[17] Lin J, Hunkapiller AA, Layton AC, Chang Y-J, Robbins KR (2013). Response of intestinal microbiota to antibiotic growth promoters in chickens. Foodborne Pathog Dis.

[18] Dumonceaux TJ, Hill JE, Hemmingsen SM, Van Kessel AG (2006). Characterization of intestinal microbiota and response to dietary virginiamycin supplementation in the broiler chicken. Appl Environ Microbiol.

[19] Danzeisen JL, Kim HB, Isaacson RE, Tu ZJ, Johnson TJ (2011). Modulations of the chicken cecal microbiome and metagenome in response to anticoccidial and growth promoter treatment. PLoS One.

[20] Sergeant MJ, Constantinidou C, Cogan TA, Bedford MR, Penn CW, Pallen MJ (2014). Extensive microbial and functional diversity within the chicken cecal microbiome. PLoS One.

[21] Stanley D, Geier MS, Hughes RJ, Denman SE, Moore RJ (2013). Highly variable microbiota development in the chicken gastrointestinal tract. PloS One.

[22] Gong J, Yu H, Liu T, Gill J, Chambers J, Wheatcroft R (2008). Effects of zinc bacitracin, bird age and access to range on bacterial microbiota in the ileum and caeca of broiler chickens. J Appl Microbiol.

[23] Looft T, Allen HK, Casey TA, Alt DP, Stanton TB (2014). Carbadox has both temporary and lasting effects on the swine gut microbiota. Antimicrobials, Resistance and. Chemotherapy.

[24] Kim HB, Borewicz K, White BA, Singer RS, Sreevatsan S, Tu ZJ (2012). Microbial shifts in the swine distal gut in response to the treatment with antimicrobial growth promoter, tylosin. Proc Natl Acad Sci.

[25] Holman DB, Chénier MR (2014). Temporal changes and the effect of subtherapeutic concentrations of antibiotics in the gut microbiota of swine. FEMS Microbiol Ecol.

[26] Guban J, Korver D, Allison G, Tannock G (2006). Relationship of dietary antimicrobial drug administration with broiler performance, decreased population levels of Lactobacillus salivarius, and reduced bile salt deconjugation in the ileum of broiler chickens. Poult Sci.

[27] Okino S, Inui M, Yukawa H (2005). Production of organic acids by Corynebacterium glutamicum under oxygen deprivation. Appl Microbiol Biotechnol.

[28] Cousin FJ, Jouan-Lanhouet S, Dimanche-Boitrel M-T, Corcos L, Jan G (2012). Milk fermented by Propionibacterium freudenreichii induces apoptosis of HGT-1 human gastric cancer cells. PLoS One.

[29] Foligné B, Deutsch S-M, Breton J, Cousin FJ, Dewulf J, Samson M (2010). Promising immunomodulatory effects of selected strains of dairy propionibacteria as evidenced in vitro and in vivo. Appl Environ Microbiol.

[30] Jan G, Belzacq A, Haouzi D, Rouault A, Metivier D, Kroemer G (2002). Propionibacteria induce apoptosis of colorectal carcinoma cells via short-chain fatty acids acting on mitochondria. Cell Death Differ.

[31] Kekkonen RA, Lummela N, Karjalainen H, Latvala S, Tynkkynen S, Järvenpää S (2008). Probiotic intervention has strain-specific anti-inflammatory effects in healthy adults. World J Gastroenterol.

[32] El-Nezami H, Mykkanen H, Kankaanpaa P, Salminen S, Ahokas J (2000). Ability of Lactobacillus and Propionibacterium strains to remove aflatoxin B1 from the chicken duodenum. J Food Prot.

[33] Kabel MA, Kortenoeven L, Schols HA, Voragen AG (2002). In vitro fermentability of differently substituted xylo-oligosaccharides. J Agric Food Chem.

[34] Chung Y-C, Hsu C-K, Ko C-Y, Chan Y-C (2007). Dietary intake of xylooligosaccharides improves the intestinal microbiota, fecal moisture, and pH value in the elderly. Nutr Res.

[35] Campbell JM, Fahey GC, Wolf BW (1997). Selected indigestible oligosaccharides affect large bowel mass, cecal and fecal short-chain fatty acids, pH and microflora in rats. J Nutr.

[36] Hsu C-K, Liao J-W, Chung Y-C, Hsieh C-P, Chan Y-C (2004). Xylooligosaccharides and fructooligosaccharides affect the intestinal microbiota and precancerous colonic lesion development in rats. J Nutr.

[37] Eeckhaut V, Van Immerseel F, Dewulf J, Pasmans F, Haesebrouck F, Ducatelle R (2008). Arabinoxylooligosaccharides from wheat bran inhibit Salmonella colonization in broiler chickens. Poult Sci.

[38] Duncan SH, Louis P, Flint HJ (2004). Lactate-utilizing bacteria, isolated from human feces, that produce butyrate as a major fermentation product. Appl Environ Microbiol.

[39] Elferink SJO, Krooneman J, Gottschal JC, Spoelstra SF, Faber F, Driehuis F (2001). Anaerobic conversion of lactic acid to acetic acid and 1, 2-propanediol by Lactobacillus buchneri. Appl Environ Microbiol.

[40] Gong J, Si W, Forster RJ, Huang R, Yu H, Yin Y (2007). 16S rRNA gene-based analysis of mucosa-associated bacterial community and phylogeny in the chicken gastrointestinal tracts: from crops to ceca. FEMS Microbiol Ecol.

[41] Sekelja M, Rud I, Knutsen S, Denstadli V, Westereng B, Næs T (2012). Abrupt temporal fluctuations in the chicken fecal microbiota are explained by its gastrointestinal origin. Appl Environ Microbiol.

[42] Videnska P, Sisak F, Havlickova H, Faldynova M, Rychlik I (2013). Influence of Salmonella enterica serovar Enteritidis infection on the composition of chicken cecal microbiota. BMC Veter Res.

[43] Wielen P, Keuzenkamp D, Lipman L, Knapen F, Biesterveld S (2002). Spatial and temporal variation of the intestinal bacterial community in commercially raised broiler chickens during growth. Microb Ecol.

[44] van Dongen WF, White J, Brandl HB, Moodley Y, Merkling T, Leclaire S (2013). Age-related differences in the cloacal microbiota of a wild bird species. BMC Ecol.

[45] Wise M, Siragusa G (2007). Quantitative analysis of the intestinal bacterial community in one-to three-week-old commercially reared broiler chickens fed conventional or antibiotic-free vegetable-based diets. J Appl Microbiol.

[46] Lu J, Idris U, Harmon B, Hofacre C, Maurer JJ, Lee MD (2003). Diversity and succession of the intestinal bacterial community of the maturing broiler chicken. Appl Environ Microbiol.

[47] Kim M, Morrison M, Yu Z (2011). Evaluation of different partial 16S rRNA gene sequence regions for phylogenetic analysis of microbiomes. J Microbiol Methods.

[48] Caporaso JG, Kuczynski J, Stombaugh J, Bittinger K, Bushman FD, Costello EK (2010). QIIME allows analysis of high-throughput community sequencing data. Nat Methods.

[49] Reeder J, Knight R (2010). Rapid denoising of pyrosequencing amplicon data: exploiting the rank-abundance distribution. Nat Methods.

[50] Edgar RC, Haas BJ, Clemente JC, Quince C, Knight R (2011). UCHIME improves sensitivity and speed of chimera detection. Bioinformatics.

[51] Fu L, Niu B, Zhu Z, Wu S, Li W (2012). CD-HIT: accelerated for clustering the next-generation sequencing data. Bioinformatics.

[52] Caporaso JG, Bittinger K, Bushman FD, DeSantis TZ, Andersen GL, Knight R (2010). PyNAST: a flexible tool for aligning sequences to a template alignment. Bioinformatics.

[53] Wang Q, Garrity GM, Tiedje JM, Cole JR (2007). Naive Bayesian classifier for rapid assignment of rRNA sequences into the new bacterial taxonomy. Appl Environ Microbiol.

[54] Bokulich NA, Subramanian S, Faith JJ, Gevers D, Gordon JI, Knight R (2012). Quality-filtering vastly improves diversity estimates from Illumina amplicon sequencing. Nat Methods.

[55] Clarke KR (1993). Non-parametric multivariate analyses of changes in community structure. Aust J Ecol.

[56] Segata N, Izard J, Waldron L, Gevers D, Miropolsky L, Garrett WS (2011). Metagenomic biomarker discovery and explanation. Genome Biol.

