# Supplementary material for: Xylo-oligosaccharides and virginiamycin differentially modulate gut microbial composition in chickens
Source: Microbiome. 2015 Apr 10;3:15. doi: 10.1186/s40168-015-0079-4 (PMC4396176; doi:10.1186/s40168-015-0079-4)
Supplement: Additional file 1: — Supplementary data. Table S1. Statistical comparison of alpha diversities between treatments in the ileal and cecal microbiota. A nonparametric t-test was run in QIIME to compare the alpha diversities using the default number of Monte Carlo permutations (999) and the greatest rarefaction depth. Cells shaded with light blue (upper right section) shows the P values for the number of observed species while those shaded with light yellow (lower left section) shows the P values of phylogenetic diversity comparison. Table S2. Statistical comparison of alpha diversities between ages in the ilea and cecal microbiota. A nonparametric two sample t-test was run in QIIME to compare the alpha diversities using the default number of Monte Carlo permutations (999) and the greatest rarefaction depth. Cells shaded with light blue (upper right section) shows the P values for the number of observed species while those shaded with light yellow (lower left section) shows the P values of phylogenetic diversity comparison. Significant P values are bolded. Table S3. Ingredient composition (g/kg) of experimental diets. Figure S1. The ileal and cecal microbial composition on days 15, 25, and 35. The bar charts indicate the relative abundance (%) of bacterial phyla (A) and the dominant (>0.5% of sequences) bacterial families and genera (B) in the ileum and cecal microbiota of chickens. Classification is according to the RDP trained on the Greengenes database with a minimum confidence score of 0.8. For each habitant, ileal and cecal contents of 16 birds were collected at 15, 25, and 35 days old. [file 40168_2015_79_MOESM1_ESM.docx]

**Table S1.** Statistical comparison of alpha diversities between treatments in the ileal and cecal microbiota. A nonparametric t-test was run in QIIME to compare the alpha diversities using the default number of Monte Carlo permutations (999) and the greatest rarefaction depth. Cells shaded with light blue (upper right section) shows the p-values for the number of observed species while those shaded with light yellow (lower left section) shows the p-values of phylogenetic diversity comparison.

| Observed Species | | | | | | | | |
| --- | --- | --- | --- | --- | --- | --- | --- | --- |
|  | Ileum | | | | Cecum | | | |
| Treatments | CTL | VIRG | LXOS | HXSO | CTL | VIRG | LXOS | HXOS |
| CTL | 1 | 0.24 | 0.33 | 0.08 | 1 | 1 | 1 | 1 |
| VIRG | 0.16 | 1 | 1 | 0.87 | 1 | 1 | 1 | 1 |
| LXOS | 0.15 | 1 | 1 | 1 | 1 | 1 | 1 | 1 |
| HXOS | 0.06 | 0.79 | 1 | 1 | 1 | 1 | 1 | 1 |
| Phylogenetic Diversity | | | | | | | | |

**Table S2.** Statistical comparison of alpha diversities between ages in the ilea and cecal microbiota. A nonparametric two sample t-test was run in QIIME to compare the alpha diversities using the default number of Monte Carlo permutations (999) and the greatest rarefaction depth. Cells shaded with light blue (upper right section) shows the p-values for the number of observed species while those shaded with light yellow (lower left section) shows the p-values of phylogenetic diversity comparison. Significant p-values are bolded.

| Observed Species | | | | | | |
| --- | --- | --- | --- | --- | --- | --- |
|  | Ileum | | | Cecum | | |
| Age | Day 15 | Day 25 | Day 35 | Day 15 | Day 25 | Day 35 |
| Day 15 | 1 | 1 | 1 | 1 | 1 | **0.003** |
| Day 25 | 1 | 1 | 1 | 0.73 | 1 | **0.003** |
| Day 35 | 0.66 | 1 | 1 | **0.003** | 0.003 | 1 |
| Phylogenetic Diversity | | | | | | |

**Table S3.** Ingredient composition (g/kg) of experimental diets

| Ingredient | CTL | VIRG | LXOS | HXOS |  |  |  |  |
| --- | --- | --- | --- | --- | --- | --- | --- | --- |
| Corn | 511.28 | 511.28 | 511.28 | 511.28 |  |  |  |  |
| Soya | 300.36 | 300.36 | 300.36 | 300.36 |  |  |  |  |
| Soybean meal, 48% CP | 141.64 | 141.64 | 141.64 | 141.64 |  |  |  |  |
| Phosphore | 17.22 | 17.22 | 17.22 | 17.22 |  |  |  |  |
| Calcium | 13.88 | 13.88 | 13.88 | 13.88 |  |  |  |  |
| micro debut | 5.5 | 5.5 | 5.5 | 5.5 |  |  |  |  |
| NaCL | 2.17 | 2.17 | 2.17 | 2.17 |  |  |  |  |
| Lysine | 2.16 | 2.16 | 2.16 | 2.16 |  |  |  |  |
| Filler | 2 | 1.62 | 1 | 0 |  |  |  |  |
| Methionine | 1.3 | 1.3 | 1.3 | 1.3 |  |  |  |  |
| Choline chloride | 1 | 1 | 1 | 1 |  |  |  |  |
| Sodium carbonate | 1 | 1 | 1 | 1 |  |  |  |  |
| XOS | 0 | 0 | 1 | 2 |  |  |  |  |
| Threonine | 0.48 | 0.48 | 0.48 | 0.48 |  |  |  |  |
| Virginiamycin | 0 | 0.38 | 0 | 0 |  |  |  |  |
| Vitamin Mixture 10% | 0.01 | 0.01 | 0.01 | 0.01 |  |  |  |  |
| Calculated analysis |  |  |  |  |  |  |  |  |
| ME, kcal/kg | | | | | 3029 | 3029 | 3029 | 3029 |
| Protein, g/kg | 23 | 23 | 23 | 23 |  |  |  |  |
| Lysine, g/kg | 1.43 | 1.43 | 1.43 | 1.43 |  |  |  |  |
| Methionine, g/kg | 0.51 | 0.51 | 0.51 | 0.51 |  |  |  |  |
| Crude Fat, g/kg | 4.45 | 4.45 | 4.45 | 4.45 |  |  |  |  |
|  |  |  |  |  |  |  |  |  |

CTL: Control diet without any antibiotic or prebiotic; VIRG: Control diet supplemented with 16.5 mg virginiamycin; LXOS: Control diet supplemented with 1g xylo-oligosaccharides/kg feed; HXOS: Control diet supplemented with 2g xylo-oligosaccharides/kg fee

**Figure S1.** The ileal and cecal microbial composition on day 15, 25 and 35. The bar charts indicate the relative abundance (%) of bacterial phyla (**A**) and the dominant (>0.5% of sequences) bacterial families and genera (**B**) in the ileum and cecal microbiota of chickens. Classification is according to the RDP trained on the Greengenes database with a minimum confidence score of 0.8. For each habitant, ileal and cecal contents of 16 birds were collected at 15, 25 and 35 days old.

**
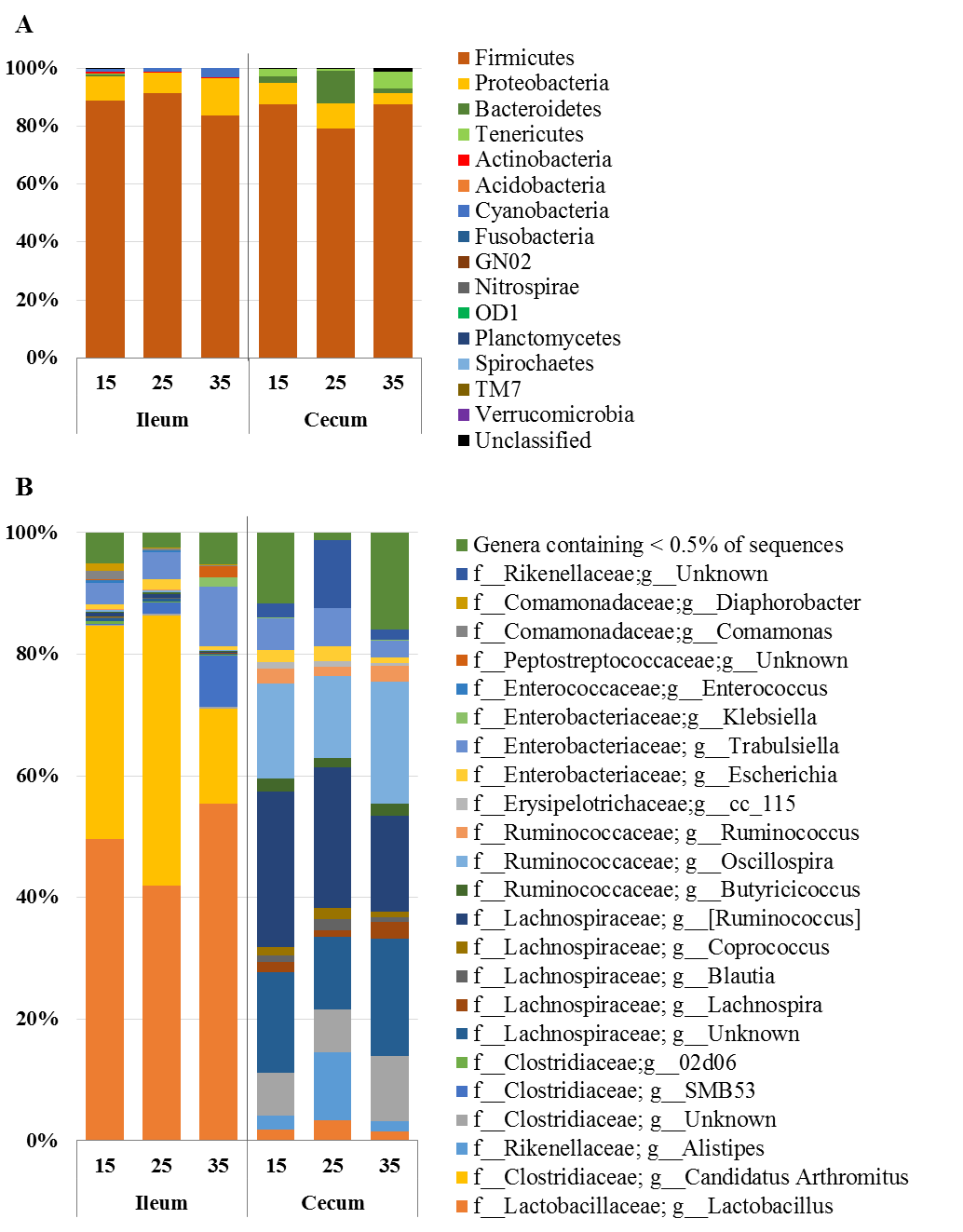
**
